# Supplementary material for: TSEBRA: transcript selector for BRAKER
Source: BMC Bioinformatics. 2021 Nov 25;22:566. doi: 10.1186/s12859-021-04482-0 (PMC8620231; doi:10.1186/s12859-021-04482-0)
Supplement: Supplementary file 1 — Additional file 1. Tables (S1–S6). The supplementary tables include a list of all genomes and annotation versions used in this paper, additional results, hyperparameters and statistical significance tests. [file 12859_2021_4482_MOESM1_ESM.pdf]

Supplementary Tables

Table S1 List of all genomes and annotation versions used for the experiments. The species with * are the model species used for the comparison of TSEBRA and EVM.

Species	Annotation version	Genome size (Mb)	Number of genes
*Arabidopsis thaliana**	Tair Araport 11 (Jun 2016)	119	27,445
Bombus terrestris	NCBI Annotation Release 102(Apr 2017)	249	10,581
*Caenorhabditis elegans**	WormBase WS271 (May 2019)	100	20,172
Danio rerio	Ensembl GRCz11.96 (May 2019)	1345	25,254
*Drosophila melanogaster**	FlyBase R6.28 (Jun 2019)	138	13,929
Medicago truncatula	MtrunA17r5.0-ANR-EGN-r1.6 (Feb 2019)	430	44,464
Parasteatoda tepidariorum	NCBI Annotation Release 101 (May 2017)	1445	18,602
Populus trichocarpa	JGI Ptrichocarpa 533 v4.1 (Nov2019)	389	34,488
Rhodnius prolixus	VectorBase RproC3.3 (Oct 2017)	707	15,061
Tetraodon nigroviridis	TETRAODON8.99 (Nov 2019)	359	19,589
Xenopus tropicalis	NCBI Annotation Release 104 (Apr 2019)	1449	21,821

Table S2 F1-score on CDS, transcript, and gene level for BRAKER1 (RNA-seq hints), BRAKER2 (protein hints), TSEBRA_EVM, EVM using comparable evidence, and TSEBRA with hints generated by the BRAKER runs. The methods were run three times for each species, allowing different minimum evolutionary distances ((i) species excluded, (ii) family excluded or (iii) order excluded) for the proteins used as support. A different set of genome partitions, each totaling 90% of the genome size from a species, was sampled for the evaluation of all methods for each model species and test setting.

	**CDS level F1-score**				
	BRAKER1	BRAKER2	EVM	TSEBRA_EVM	TSEBRA
A. tha.(i)	81.67	84.62	85.10	**87.70**	87.34
A. tha.(ii)	81.67	84.00	84.07	86.46	**86.93**
A. tha.(iii)	81.87	84.01	84.41	86.21	**86.90**
C. ele.(i)	85.76	87.26	88.15	88.03	**88.27**
C. ele.(ii)	85.83	80.91	**86.46**	85.05	84.46
C. ele.(iii)	85.87	81.13	**86.14**	85.13	84.48
D. mel.(i)	79.88	84.03	83.74	85.37	**85.71**
D. mel.(ii)	79.20	79.86	80.78	81.81	**82.95**
D. mel.(iii)	79.82	76.79	79.67	79.89	**81.66**
	**Transcript level F1-score**				
	BRAKER1	BRAKER2	EVM	TSEBRA_EVM	TSEBRA
A. tha.(i)	53.52	60.25	61.01	**64.92**	64.05
A. tha.(ii)	53.48	57.02	56.63	61.97	**62.14**
A. tha.(iii)	53.78	56.63	57.32	61.35	**62.00**
C. ele.(i)	53.44	55.13	57.41	59.66	**60.54**
C. ele.(ii)	53.36	42.52	53.79	54.58	**56.09**
C. ele.(iii)	53.30	42.71	52.76	54.46	**55.94**
D. mel.(i)	51.44	58.50	57.86	**62.26**	61.61
D. mel.(ii)	51.22	50.98	52.23	56.37	**57.01**
D. mel.(iii)	51.33	46.94	49.90	53.76	**55.18**
	**Gene level F1-score**				
	BRAKER1	BRAKER2	EVM	TSEBRA_EVM	TSEBRA
A. tha.(i)	65.20	75.03	75.09	**81.45**	81.15
A. tha.(ii)	65.22	71.08	70.07	78.92	**79.77**
A. tha.(iii)	65.51	70.58	70.88	78.35	**79.69**
C. ele.(i)	63.25	67.18	69.37	73.80	**76.31**
C. ele.(ii)	63.12	52.15	65.18	69.30	**70.92**
C. ele.(iii)	63.13	52.29	63.98	68.90	**70.78**
D. mel.(i)	64.56	75.31	74.75	80.66	**81.46**
D. mel.(ii)	64.12	66.08	67.81	74.46	**76.07**
D. mel.(iii)	64.44	61.25	64.94	71.34	**73.93**

Statistical significance testing

For all test settings, we compared TSEBRA in default mode to to the gene sets of BRAKER1, BRAKER2, and EVM on transcript and CDS level pairwise, see Table S5. For *A. thal*, *C. ele.*, and *D. mel.*, we compared TSEBRA_EVM with all methods, other than TSEBRA, see Table S6.

We used McNemar's test to determine if there was a significant difference between

Table S3 Weights for each input source used by the EvidenceModeler for the comparison with TSEBRA_EVM for each model species and all test settings.

	BRAKER	BRAKER2	RNA-seq	Protein
A. tha.(i)	30	6750	22	22
A. tha.(ii)	30	6750	7	6750
A. tha.(iii)	30	6750	22	22
C. ele.(i)	30	480	900	480
C. ele.(ii)	30	22	60	7
C. ele.(iii)	30	22	60	22
D. mel.(i)	30	6750	45	6750
D. mel.(ii)	30	480	6750	0
D. mel.(iii)	30	22	60	30

Table S4 Hyperparameters used by TSEBRA_EVM for the comparison with TSEBRA_EVM for each model species and all test settings. The hyperparameter sets are described in Implementation.

	**Evidence source weight**		**Low Evidence support threshold**		**Transcript score threshold**			
	Protein (w_P)	RNA-seq (w_R)	Intron	Start/stop-codon	ϵ_1	ϵ_2	ϵ_3	ϵ_4
A. tha.(i)	15.0	10.0	0.75	1.0	0.0	0.0	20.0	0.0
A. tha.(ii)	10.0	0.5	0.75	1.0	0.0	0.0	10.0	0.0
A. tha.(iii)	5.5	10.0	0.5	1.0	0.0	0.5	30.0	2.5
C. ele.(i)	5.5	15.0	0.0	0.0	0.0	1.0	10.0	2.5
C. ele.(ii)	0.5	10.0	0.0	0.0	0.0	0.5	20.0	10.0
C. ele.(iii)	0.5	10.0	0.0	0.0	0.0	0.5	10.0	15.0
D. mel.(i)	1.0	0.5	0.875	0.5	0.0	0.0	5.0	0.0
D. mel.(ii)	5.5	0.75	0.25	1.0	0.0	0.5	20.0	10.0
D. mel.(iii)	5.5	1.0	0.25	0.5	0.0	0.5	15.0	15.0

the methods in the frequency of correctly predicted and missing transcripts/CDS from the reference annotation by the two methods. Therefore, we assumed TSEBRA and the compared method as paired tests with the transcripts/CDS of the reference annotation as the sample. We interpreted this test as a comparison of sensitivity between two methods. To compare the specificity between TSEBRA and the other methods in a similar way, we performed the chi-square test of homogeneity with all pairs. Here, the results of each method are considered binary experiments: Is a predicted feature correct, i.e. also in the reference annotation or not? The test checks the null hypothesis that these underlying distributions for the two tools have the same probability of being correct.

Table S5 P-values of statistical tests that compare the differences of sensitivity and specificity from TSEBRA_EVM to BRAKER1, BRAKER2, and EVM. We used McNemar's test to compare the sensitivity (Sn) and the chi-square test of homogeneity to compare the specificity (Sp). Green cells mark a sensitivity/specificity where TSEBRA has a higher or equal value than the compared method and red marks the ones where TSEBRA has a lower value.

CDS level

	BRAKER1		BRAKER2		EVM	
	Sn	Sp	Sn	Sp	Sn	Sp
A. tha. (i)	< 0.0001	< 0.0001	0.0007	< 0.0001	< 0.0001	< 0.0001
A. tha. (ii)	< 0.0001	< 0.0001	0.6038	< 0.0001	< 0.0001	< 0.0001
A. tha. (iii)	< 0.0001	< 0.0001	0.0020	< 0.0001	< 0.0001	< 0.0001
B. ter.	< 0.0001	< 0.0001			< 0.0001	
C. ele. (i)	< 0.0001	< 0.0001	0.0002	< 0.0001	< 0.0001	< 0.0001
C. ele. (ii)	< 0.0001	< 0.0001	< 0.0001	< 0.0001	< 0.0001	< 0.0001
C. ele. (iii)	< 0.0001	< 0.0001	< 0.0001	< 0.0001	< 0.0001	< 0.0001
D. rer.	< 0.0001	0.0045			< 0.0001	
D. mel. (i)	0.0006	< 0.0001	< 0.0001	< 0.0001	< 0.0001	< 0.0001
D. mel. (ii)	< 0.0001	< 0.0001	< 0.0001	< 0.0001	0.7087	< 0.0001
D. mel. (iii)	< 0.0001	< 0.0001	< 0.0001	< 0.0001	< 0.0001	< 0.0001
M. tru.	< 0.0001	< 0.0001			< 0.0001	
P. tep.	< 0.0001	< 0.0001			< 0.0001	
P. tri.	< 0.0001	< 0.0001			< 0.0001	
R. pro.	< 0.0001	0.8220			< 0.0001	
T. nig.	< 0.0001	< 0.0001			< 0.0001	
X. tro.	< 0.0001	< 0.0001			< 0.0001	

Transcript level

	BRAKER1		BRAKER2		EVM	
	Sn	Sp	Sn	Sp	Sn	Sp
A. tha. (i)	< 0.0001	< 0.0001	< 0.0001	< 0.0001	< 0.0001	< 0.0001
A. tha. (ii)	< 0.0001	< 0.0001	< 0.0001	< 0.0001	< 0.0001	< 0.0001
A. tha. (iii)	< 0.0001	< 0.0001	< 0.0001	< 0.0001	< 0.0001	< 0.0001
B. ter.	< 0.0001	< 0.0001			< 0.0001	
C. ele. (i)	< 0.0001	< 0.0001	< 0.0001	< 0.0001	< 0.0001	0.0570
C. ele. (ii)	< 0.0001	< 0.0001	< 0.0001	< 0.0001	< 0.0001	< 0.0001
C. ele. (iii)	< 0.0001	< 0.0001	< 0.0001	< 0.0001	< 0.0001	< 0.0001
D. rer.	< 0.0001	< 0.0001			< 0.0001	
D. mel. (i)	< 0.0001	< 0.0001	0.1403	< 0.0001	< 0.0001	< 0.0001
D. mel. (ii)	< 0.0001	< 0.0001	< 0.0001	< 0.0001	< 0.0001	< 0.0001
D. mel. (iii)	0.1250	< 0.0001	< 0.0001	< 0.0001	< 0.0001	< 0.0001
M. tru.	< 0.0001	< 0.0001			< 0.0001	
P. tep.	< 0.0001	< 0.0001			< 0.0001	
P. tri.	< 0.0001	< 0.0001			< 0.0001	
R. pro.	< 0.0001	< 0.0001			< 0.0001	
T. nig.	< 0.0001	< 0.0001			< 0.0001	
X. tro.	< 0.0001	< 0.0001			< 0.0001	

Table S6 P-values of statistical tests that compare the differences of sensitivity and specificity from TSEBRA_EVM to BRAKER1, BRAKER2, and EVM. We used McNemar's test to compare the sensitivity (Sn) and the chi-square test of homogeneity to compare the specificity (Sp). Green cells mark a sensitivity/specificity where TSEBRA_EVM has a higher or equal value than the compared method and red marks the ones where TSEBRA_EVM has a lower value.

CDS level

	BRAKER1		BRAKER2		EVM	
	Sn	Sp	Sn	Sp	Sn	Sp
A. tha. (i)	< 0.0001	< 0.0001	< 0.0001	< 0.0001	< 0.0001	< 0.0001
A. tha. (ii)	< 0.0001	< 0.0001	< 0.0001	< 0.0001	< 0.0001	< 0.0001
A. tha. (iii)	< 0.0001	< 0.0001	< 0.0001	< 0.0001	< 0.0001	< 0.0001
C. ele. (i)	< 0.0001	< 0.0001	< 0.0001	0.0263	< 0.0001	< 0.0001
C. ele. (ii)	< 0.0001	< 0.0001	< 0.0001	0.0463	< 0.0001	< 0.0001
C. ele. (iii)	< 0.0001	< 0.0001	< 0.0001	0.2357	< 0.0001	< 0.0001
D. mel. (i)	0.7665	< 0.0001	< 0.0001	< 0.0001	< 0.0001	< 0.0001
D. mel. (ii)	< 0.0001	< 0.0001	< 0.0001	< 0.0001	< 0.0001	< 0.0001
D. mel. (iii)	< 0.0001	< 0.0001	< 0.0001	< 0.0001	< 0.0001	< 0.0001

Transcript level

	BRAKER1		BRAKER2		EVM	
	Sn	Sp	Sn	Sp	Sn	Sp
A. tha. (i)	< 0.0001	< 0.0001	< 0.0001	< 0.0001	< 0.0001	< 0.0001
A. tha. (ii)	< 0.0001	< 0.0001	< 0.0001	< 0.0001	< 0.0001	< 0.0001
A. tha. (iii)	< 0.0001	< 0.0001	< 0.0001	< 0.0001	< 0.0001	< 0.0001
C. ele. (i)	< 0.0001	< 0.0001	< 0.0001	< 0.0001	< 0.0001	< 0.0001
C. ele. (ii)	0.0002	0.0007	< 0.0001	< 0.0001	< 0.0001	< 0.0001
C. ele. (iii)	0.6269	< 0.0001	< 0.0001	< 0.0001	< 0.0001	< 0.0001
D. mel. (i)	< 0.0001	< 0.0001	< 0.0001	< 0.0001	< 0.0001	< 0.0001
D. mel. (ii)	< 0.0001	< 0.0001	< 0.0001	< 0.0001	< 0.0001	< 0.0001
D. mel. (iii)	0.0002	< 0.0001	< 0.0001	< 0.0001	< 0.0001	0.0100
